# Pathologic Splenic Rupture in a Patient with Follicular Lymphoma

**DOI:** 10.4084/MJHID.2011.051

**Published:** 2011-11-10

**Authors:** A.P. Dayama, R. Kapoor, J. Dass, G. Singh, M. Mahapatra, H.P. Pati

**Affiliations:** Department of Hematology, All India Institute of Medical Sciences, New Delhi, India

## Abstract

A middle aged man presented with abdominal pain and fever, with progressive dyspnea for the past one week. He had generalized lymphadenopathy with hepatosplenomegaly and a left sided pleural effusion on admission. Further evaluation revealed that he had lymphocytosis on peripheral blood. He then developed increasing abdominal pain and fall in hemoglobin which was confirmed on imaging to be due to a splenic rupture and he underwent a splenectomy. The diagnosis on lymph node biopsy and peripheral blood immunophenotyping was grade 1 follicular lymphoma. He has completed his 6 cycles of chemotherapy (R-CVP) and is on maintenance rituximab and doing well. The case highlights the fact that splenic rupture can even be caused by indolent lymphomas.

Follicular lymphoma (FL) is the most common indolent non Hodgkin’s lymphoma (NHL).^1^ It presents primarily with widespread disease which may be asymptomatic and involves the bone marrow in around 40% of patients.^2^ Although the disease is widespread at presentation the incidence of complications such as splenic rupture which are usually seen with other aggressive lymphomas is rare.

A 48 year old male presented to the emergency with complaints of increasing abdominal distension and pain. He also had a history of fever and breathlessness for the past 1 week. There was no history of weight loss, skin rash, bleeding tendencies, bone/joint pains or altered bowel/urinary habits. On examination he had generalized lymphadenopathy (bilateral cervical, axillary, inguinal), hepatosplenomegaly and decreased breath sounds over left hemithorax. Rest of the examination was essentially normal. Investigations revealed hemoglobin 11.7g/dL, total leucocyte count -62,820/ μL, platelet count- 169000/μL. The blood smear showed few smudge cells with mature lymphocytosis (N6 L93, M1) with no malarial parasite and a reticulocyte count of 2%. An ultrasound of the abdomen and chest showed hepatosplenomegaly and small left sided pleural effusion. He developed worsening of his symptoms with increased breathlessness and pain in abdomen after admission. Chest X ray showed increasing pleural effusion on the left side. However in view of progressive worsening of abdominal pain in the next day, a repeat chest and abdominal CT was done, which showed left pleural effusion with hepatosplenomegaly and multiple lymph nodes in neck, mediastinum, bilateral axillae, retroperitoneal region. There was a hypodense lesion in spleen suggestive of splenic rupture (Figure 1A). He also developed fall in his hemoglobin from 10.4 to 6.3 g/dL in 3 days with severe abdominal pain for which he underwent emergency splenectomy. The bone marrow aspirate showed near total replacement by lymphocytes whereas the biopsy revealed diffuse infiltration by mature lymphocytes. The immunophenotyping on peripheral blood was positive for CD20, CD22, CD23, CD19, FMC7, CD79b and lambda restriction. It was negative for CD5/23 co-exp, CD5, CD10, CD38, Zap 70, CD2, CD3, CD34, CD25, CD11c, CD103. The LDH level was 385 U/dL and the serology for HIV-1 & 2, HBsAg, Anti-HCV was negative. An axillary lymph node biopsy was reported as grade 1 Follicular Lymphoma (Figure 1B and 1C). Immunohistochemistry of lymphnode biopsy showed positive for CD 20, CD10 and BCL-2, negative for Cyclin D1. The splenectomy specimen also showed involvement by follicular lymphoma. The patient then received chemotherapy (R-CVP) and was discharged in a stable condition with Hb 11.1 gm/dL, TLC 9490/μL, Plt 835000/μL. The patient has finished 6 cycles of R-CVP and is on 3 monthly maintenance Rituximab therapy and is doing well.

Follicular lymphoma frequently involves spleen but incidence of spontaneous splenic rupture is very rare. A variety of other causes including acute leukemias, chronic leukemias, Hodgkin’s disease and NHL have been reported to cause spontaneous splenic rupture.^3^ Pathologic rupture of spleen may be caused by the infiltration of the splenic capsule by malignant cells leading to its congestion, splenic infarction and possibly co-existent coagulation abnormalities leading to subcapsular hematomas and capsular rupture.^4^ After searching the MEDLINE database, we came across only 1 series by Howard et al who have described a total of 16 cases of FL involving the spleen among which 3 underwent splenectomy for splenic rupture. In their series, 12/16 patients were diagnosed as FL on the splenectomy specimen including the 3 cases presenting with splenic rupture while in the rest 4 cases, the diagnosis was established before splenectomy.^5^ Splenic rupture has been seen in cases of mantle cell lymphoma^6^ and only rarely in other low grade lymphomas such as Waldenstrom macroglobulinemia.^7^ This highlights the fact that even though rare the possibility of indolent lymphomas such as FL should be kept in mind in a patient who has rupture of the spleen.

## Figures and Tables

**Figure 1:**
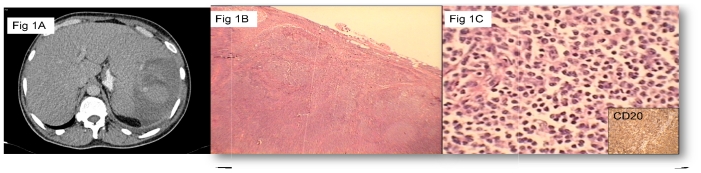
**1A:** CECT abdomen shows splenic rupture with hemoperitoneum. **1B:** Low power view of lymph node showing follicular pattern (X100). **1C:** High power view showing a predominantly centrocytic population and the inset shows CD20 positivity (X400).
